# Outcomes of 11 cats with Achilles tendon (AT) rupture repair treated with a synthetic ultra‐high‐molecular‐weight polyethylene (UHMWPE)

**DOI:** 10.1111/vsu.70107

**Published:** 2026-04-08

**Authors:** Clara Bohin, Guillaume Reinsch, Bertrand Vedrine, Guillaume Ragetly, Philippe Buttin, Frans J. Venter, Jonathan Deacon, Fabrice Bernard

**Affiliations:** ^1^ CHV Saint‐Martin Allonzier‐la‐Caille France; ^2^ Clinique TouraineVet Rochecorbon France; ^3^ CHV Vivanima Rouen France; ^4^ CHV Fregis Paris France; ^5^ VetEchoChir Villaz France; ^6^ Tropical Vet, North Shore Townsville Clinic Burdell Australia; ^7^ Moorview Referrals Northumberland UK

## Abstract

**Objective:**

To report the outcomes of cats treated for Achilles tendon (AT) rupture with a recently commercialized synthetic ultra‐high‐molecular‐weight polyethylene (UHMWPE) implant and no transarticular fixation.

**Study design:**

Retrospective multicenter study.

**Animals:**

A total of 11 cats (13 limbs).

**Methods:**

Medical records from seven veterinary centers were reviewed for cats undergoing AT repair with the Novaten implant between 2021 and 2024. Feline musculoskeletal pain index (FMPI) was used for long‐term assessment. Lameness, posture, and pain were assessed at perioperative (0–3 months), short‐term (3–6 months), and long‐term (>12 months) intervals. Statistical analysis used non‐parametric tests.

**Results:**

A total of 13 limbs in 11 cats met the inclusion criteria. All injuries involved the tendino‐osseous junction, with 7/13 (53.8%) complete ruptures. No intraoperative complications occurred. Postoperative external coaptation was used in 9/13 (69.2%) limbs (4/13 rigid splints, 5/13 soft padded) for a median duration of 6 weeks (range, 0–8 weeks). One calcaneal fracture due to implant malposition and one surgical site infection were reported at short‐term follow‐up. Long‐term follow‐up (median, 18.5 months; range, 12.1–33) showed 11/12 limbs achieving functional recovery and normal tarsal motion.

**Conclusion:**

Repair of Achilles tendon rupture in cats using a UHMWPE implant without rigid immobilization was associated with restoration of functional limb use, normal tarsal range of motion at long‐term follow‐up, and a low incidence of postoperative complications in this case series.

**Clinical significance:**

This technique may provide a reliable surgical option for cats with AT rupture, as it may minimize the need for external fixation or external coaptation and improve short‐term recovery.

## 1 INTRODUCTION

Achilles tendon (AT) rupture is a rare orthopedic injury in cats. The common calcanean tendon, or AT, is comprised of four distinct musculotendinous structures: the paired tendons of the gastrocnemius muscle, the combined tendon of gracilis, semitendinous and biceps femoris muscles, the tendon of the superficial digital flexor muscle^1^ and the tendon of the soleus muscle, which is present only in cats.^2^ These structures converge and insert on the proximal aspect of the calcaneus.^1^ Disruption of the AT can either be complete and involve all four structures or be partial and involve only some of these structures.^1^, ^3^ Small animals with complete AT rupture show plantigrade stance with concurrent stifle extension, whereas partial ruptures lead to a lesser degree of tarsal hyperflexion.^3^ Conservative management has been attempted but the outcome is often unpredictable.^2^ Surgical repair combined with tibio‐tarsal joint immobilization has been advocated to achieve more consistent functional recovery.^2^ Several techniques have been described and include various suture patterns, the use of free fascia grafts, tendon transfers and biological or artificial implants. Reported complication rates range from 30% to 40%.^2^, ^4^, ^5^, ^6^, ^7^, ^8^ Postoperative immobilization using a calcaneo‐tibial screw or a transarticular external skeletal fixator (TESF) has been associated with up to 70% of minor complications and 40% of major complications.^2^, ^8^, ^9^

Literature on AT repair is relatively rich in dogs, but only a few studies have described the efficiency and outcome of AT repair in cats^2^, ^8^, ^10^ with only two large case series.^2^, ^8^ Surgical treatment is challenging in cats, as their tolerance of rigid postoperative joint immobilization can be poor.^8^, ^9^ Ultra‐high‐molecular‐weight polyethylene (UHMWPE) fibers have been used in orthopedic and ligament reconstruction due to their high tensile strength, resistance to fatigue, and favorable biocompatibility.^11^, ^12^, ^13^ Prior ex vivo biomechanical studies evaluating the Novaten UHMWPE implant have demonstrated high load‐to‐failure and resistance to cyclic loading, suggesting suitability for load‐bearing applications such as Achilles tendon reconstruction.^7^, ^11^, ^12^, ^13^ A previous case report described satisfactory functional clinical outcome for the repair of AT without rigid transarticular stabilization in one cat,^7^ but additional data are required to evaluate the benefits of this technique. This retrospective case series describes the short‐ and long‐term outcomes of 13 AT ruptures (acute and chronic) in 11 cats treated with this implant without rigid transarticular stabilization.

## 2 MATERIALS AND METHODS

### 2.1 Case selection criteria

Medical records of cats that underwent surgery for AT rupture at seven different veterinary institutions including four private veterinary hospitals (Saint‐Martin Veterinary Hospital, France; Fregis Veterinary Hospital, France; Moorview Referrals, England; Vivanima Veterinary Hospital, France), two referral veterinary practices (TouraineVet veterinary clinic, France; Tropical Vets—North Shore Townsville Veterinary Clinic, Australia) and one locum board‐certified surgeon practice (VetEchoChir, France) were reviewed from November 2021 to June 2024. Inclusion criteria comprised cats with a partial or complete rupture of the common calcaneal tendon repaired using the Novaten (Novetech Surgery, France) implant. Cats were included provided that perioperative medical records were sufficiently complete to document signalment, surgical technique, postoperative management, and occurrence of complications with a minimum postoperative follow‐up of 6 months. Limbs with concomitant lesions or injuries likely to interfere with implant placement or postoperative function were excluded.

### 2.2 Medical records review

Collected data included breed, age, sex, bodyweight (BW) and body condition score (BCS). Preoperative imaging (radiographs and/or ultrasound and/or MRI) was reviewed for assessment of initial injuries and indication for surgery. The pre‐ and postoperative orthopedic examination was also reviewed including gradation of lameness (according to Barnhart et al.,^12^ Table 1), muscle atrophy, initial pain on manipulation, plantigrade stance, palpation of a “gap” and type of AT lesion. Implant size, postoperative management, and the incidence and nature of complications were recorded.

**TABLE 1:** Lameness scoring scale applied to each limb at pre‐ and postoperative follow‐up intervals.^12^

Lameness score	
0	No lameness observed.
1	Mild: weightbearing lameness.
2	Moderate: weightbearing lameness with intermittent non‐weightbearing.
3	Severe: non‐weightbearing lameness with brief intermittent.
4	Non‐weightbearing lameness at all times.

### 2.3 Implant

The Novaten 2000 implant (Novetech Surgery, France) was selected for cats based on its 2000 N strength adapted to the size and weight of the animals according to the manufacturer's recommendation. The braided fibers of medical‐grade UHMWPE are the gold‐standard polymeric component in total joint replacements because of its combination of wear resistance, structural strength and biocompatibility.^7^, ^13^ The braiding provides superior mechanical and biological properties than free fibers.^14^ The implant can be inserted at the anatomical insertion site of the native tendon^7^ with its two components: a working section designed to be sutured at its proximal part to the musculotendinous junction and secured at its distal end into a bone tunnel using an interference screw; and a puller wire section allowing the insertion of the implant in bone tunnels.^7^

### 2.4 Surgical technique

Anesthetic protocols varied from one case to another depending on the assessment and choices of the anesthesiologist and/or surgeon. Antibiotic prophylaxis consisted of cefazolin 25 mg/kg IV three times daily (Cefazoline Viatris, Viatris Sante, France) or ampicillin‐sulbactam 20 mg/kg IV three times daily (Unacim, Pfizer, France) depending on the surgeon's preference and was continued at 120‐min intervals during the surgery. Analgesia included preoperative IV injection of morphine 0.3 mg/kg (Lavoisier, France) and then every 2 h during the surgery. Any pre‐existing wound was evaluated before surgery to ensure adequate skin condition and reduce the risk of SSI. Cats were positioned in dorsal, sternal or lateral recumbency depending on the surgeon's preference. The affected limb was then aseptically prepared and draped in a leg hanging technique, ensuring that no tension was applied to the musculature. A caudolateral approach to the calcaneum was made and the affected tendons were dissected from the enthesis to the musculotendinous junction. When fibrous scar tissue was observed at the tendon injury site, it was sharply excised until reaching the tendon end with normal macroscopic tendinous architecture to allow adequate healing of the tendon. The rest of the surgical technique was adapted from what is described in dogs.^7^, ^15^

**FIGURE 1:**
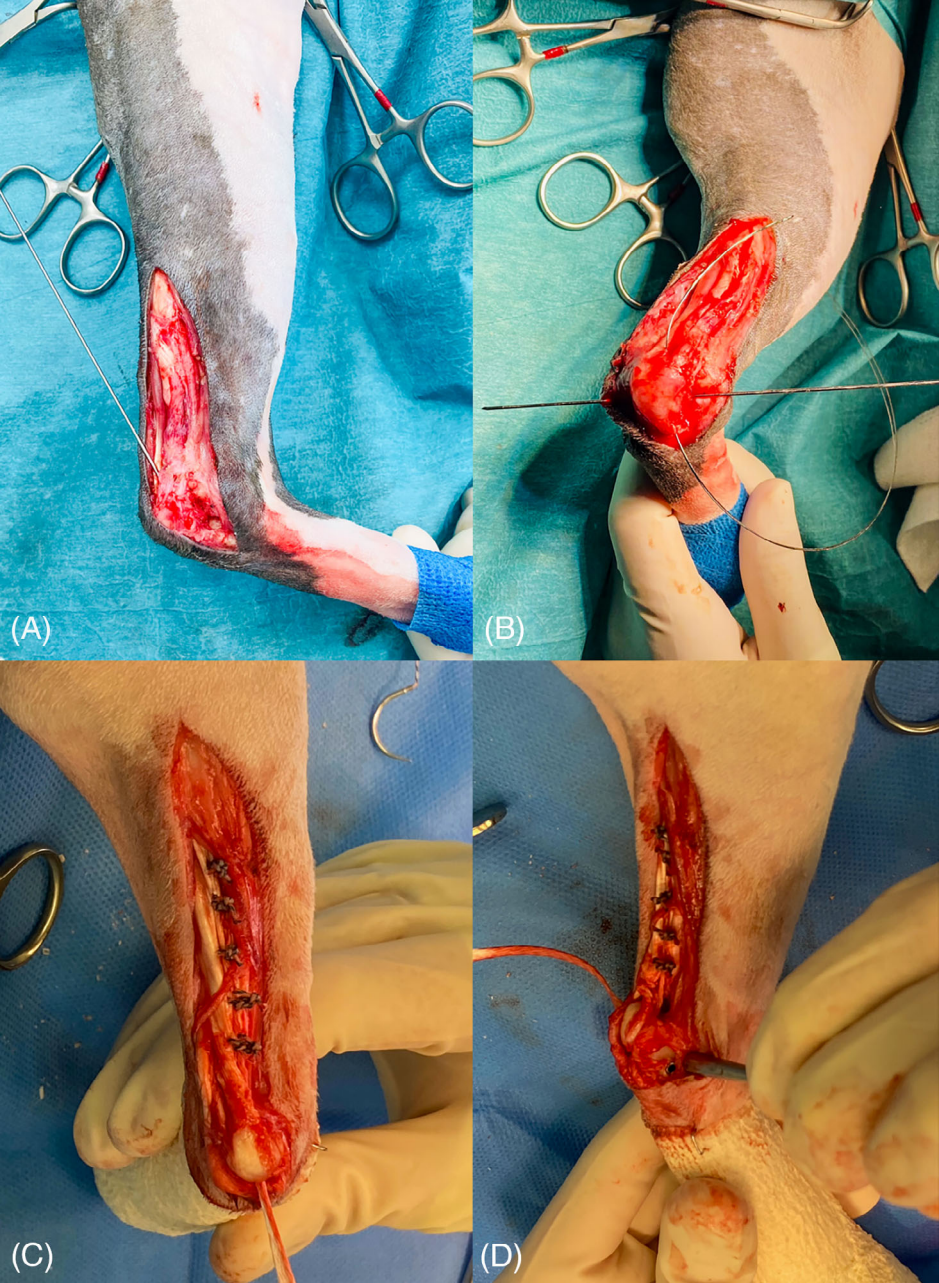
Intraoperative images. Steps of the surgery with visualization of the oblique (A) and transverse (B) bone tunnels in the calcaneum, the positioning of the implant through the oblique (C) and transverse (D) bone tunnels.

An oblique 2.5‐mm bone tunnel was drilled from the enthesis of the tendon to the plantar surface of the calcaneum with a 45° angulation using a K‐wire to secure bone tunnel position prior to overdrilling with a cannulated drill bit (Figure 1A). This tunnel was intended to pass the Novaten implant through. A second bone tunnel used for Novaten implant fixation with an interference screw was drilled perpendicular to the first one from lateral to medial at midheight of the calcaneum again using a cannulated drill bit over a K‐wire. This second tunnel was located cranially to the oblique tunnel and caudally to the talocrural joint (Figure 1B). The diameter of the tunnel was determined by the bone stock available for each patient as the diameter of the interference screw must not exceed 30% of calcaneum height to prevent bone fracture. The length of the interference screw was determined by measuring the depth of the fixation tunnel with a gauge. The fixation tunnel was then tapped or compacted with the interference screw that would be used for fixation. Both bone tunnels were flushed to remove bone debris which could damage the implant.

**FIGURE 2:**
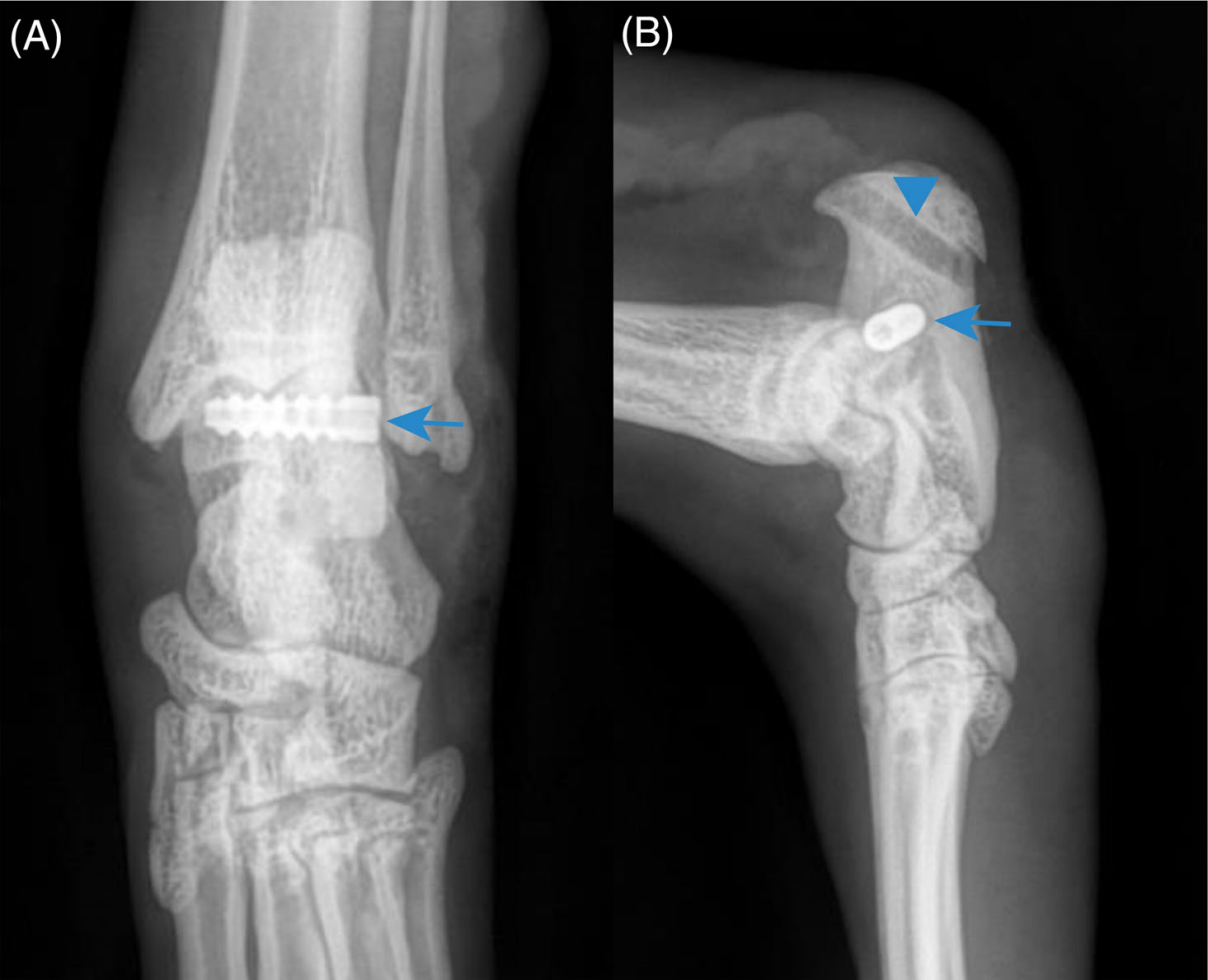
Postoperative orthogonal radiographs showing the adequate positioning of the interference screw (arrow) and oblique bone tunnel (arrowhead).

The gastrocnemius tendon was longitudinally incised on its caudal aspect after tunnel preparation, from the mid‐shaft of the gastrocnemius muscle down to the enthesis. The UHMWPE implant (Novaten 2000, Novetech Surgery, France) was unpacked and placed within the tendon incision with the extremity of the working section placed proximally to the incision. It was then secured with overlock sutures made with 2–0 UHMWPE thread (FiberTech, Novetech Surgery), as previously described.^16^ The implant was inserted in the oblique tunnel from the enthesis of the tendon to the plantar surface of the calcaneum by pulling the puller wire section using a wire loop (Figure 1C). Then the implant was inserted from lateral to medial in the distal tunnel by pulling the puller wire section. The tension was adjusted to achieve an appropriate standing angle of the tarsocrural joint. The tension of the implant was maintained using curved Kocher clamps placed at the bone tunnel exit. A 1‐mm‐diameter smooth‐ended pin was used as a guide to ensure that insertion of the cannulated interference screw follows the tunnel axis to prevent the risk of damaging the Novaten implant or fracturing the trans‐cortex. The interference screw was inserted from lateral to medial with a cannulated ratchet screwdriver (Figure 1D). The screw was left partially unscrewed, protruding by two threads, to avoid damaging the Novaten implant at the bone‐screw‐implant interface. Excess Novaten implant was trimmed to the distal tunnel exit. The surgical site was flushed thoroughly with lukewarm Hartmann's Ringers, and the tendon sheath, subcutaneous tissues and skin were closed routinely. Immediate postoperative radiographs were performed to check for the interference screw and bone tunnels position (Figure 2).

### 2.5 Postoperative management

Postoperative analgesia protocols differed across centers but typically included administration of tramadol 2–5 mg/kg orally (Tralieve, Dechra, France) for 5 to 7 days, non‐steroidal anti‐inflammatory drugs (NSAIDs) for 5 days +/− gabapentin (10–20 mg/kg orally three times daily) for 10 days. External coaptation was implemented depending on the surgeon's choice. Some limbs had no coaptation while others had coaptation up to 8 weeks with a modified Robert‐Jones with a splint. Strict activity restriction was advised for all cats for 4 to 16 weeks followed by a gradual increase in activity over the following 4 weeks prior to a reintroduction of normal activity.

### 2.6 Measures of outcomes

Recheck appointments were scheduled every week for bandage changes until removal of the splint or at 1 month postoperatively in the absence of external coaptation. At each recheck, an orthopedic examination was performed. A phone call to the owners at a minimum of 1 year postoperatively provided a feline musculoskeletal pain index (FMPI) score. The FMPI data was used to extrapolate scores for lameness, posture and pain at one year. Follow‐up was classified following Cook et al. time frames for data collection^17^ with a perioperative period (pre‐, intra‐ and postoperative) ranging from 0 to 3 months post‐surgery; a short‐term follow‐up from 3 to 6 months and a long‐term follow‐up after 12 months.^17^ Surgery was considered successful when the cat recovered full use and function of the operated hindlimb with a physiological range of motion of the affected tarsus (absence of hyper‐ or hypoextension).

### 2.7 Statistical analysis

Data are summarized using the mean, median, and range for age, bodyweight, body condition score, symptom duration before orthopedic examination, duration of postoperative coaptation, duration of activity restriction, timing of follow‐up assessments, age of the animal when the FMPI questionnaire was completed, timing of FMPI assessment, and FMPI scores. Counts and percentages were used to summarize rupture type, injury laterality, postoperative coaptation type, and lameness, posture, and pain scores. Statistical analyses were performed to evaluate changes in clinical outcomes over time and to assess the association between postoperative rigid coaptation and recovery. According to the low number of limbs, non‐parametric tests were used. Pairwise comparisons using paired sign tests were performed to assess changes in lameness, posture and pain over time. The effect of postoperative rigid coaptation on recovery rate and outcome was evaluated in two ways: (1) by comparing cats with or without rigid coaptation (yes/no) using Wilcoxon tests, and (2) by comparing the duration of rigid coaptation (none, <4 weeks, >4 weeks) using Kruskal–Wallis tests. In both analyses, lameness, pain, and posture scores were assessed at each follow‐up. The FMPI score at the final follow‐up was also analyzed using Wilcoxon and Kruskal–Wallis tests. Cases with missing data for a given time point were not included in the corresponding pairwise comparison. Statistical analyses were performed using RStudio (R version 4.4.2).

## 3 RESULTS

### 3.1 Demographics, clinical signs, and diagnostic investigations

**FIGURE 3:**
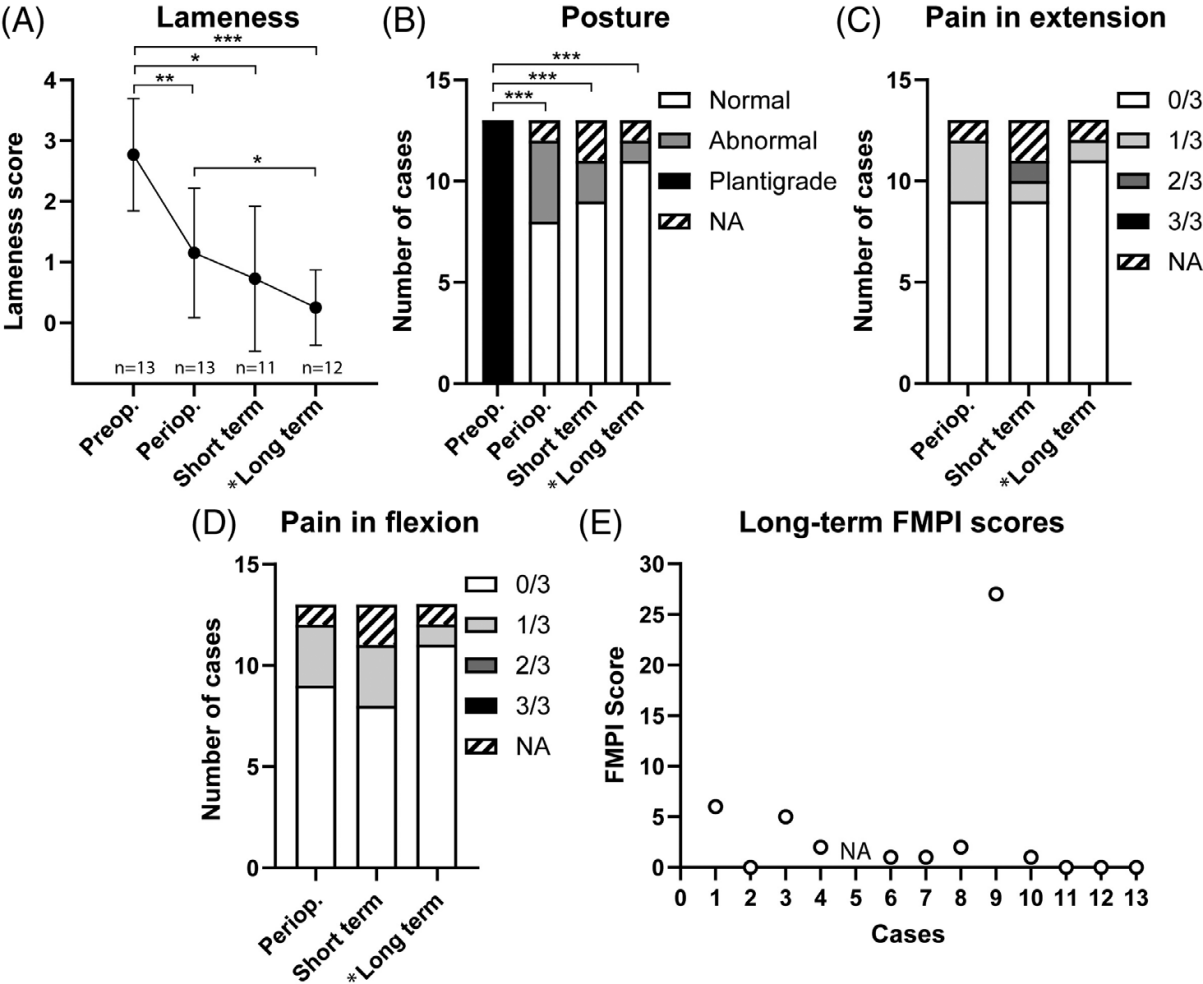
Evolution of mean lameness expressed as mean and SD (A), posture (B), pain when manipulating the joint in extension (C) or in flexion (D) over time (* scores at long‐term have been extrapolated from feline musculoskeletal pain index [FMPI] scores). *P*‐value from sign tests are set as following: *p < .05; **p < .01; ***p < .001. Only significant differences are indicated. (E) FMPI total score for each case at long‐term follow‐up.

**FIGURE 4:**
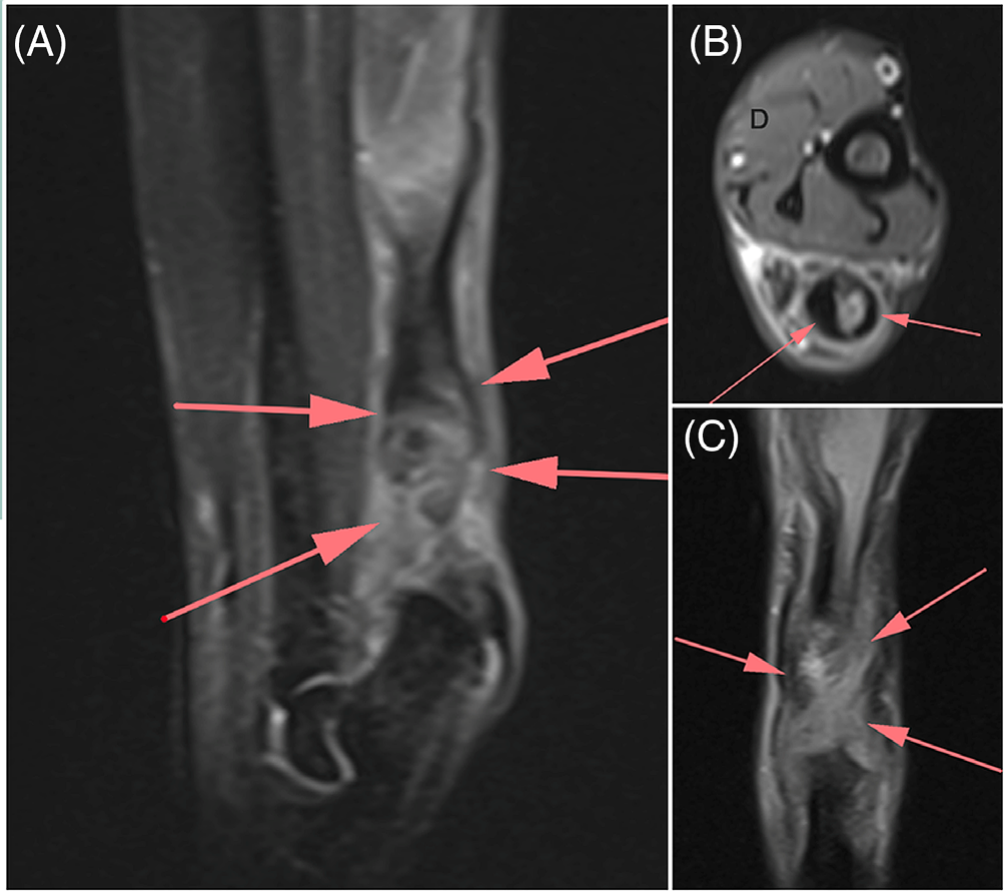
Magnetic resonance imaging (MRI) showing a complete disruption of the gastrocnemius muscle tendon at the musculotendinous junction. The flexor tendon (long and superficial) is intact.

A total of 11 cats (6 males, 5 females; all neutered) for a total of 13 limbs of AT injury met the inclusion criteria for the study. No cases were excluded due to incomplete medical records or insufficient follow‐up. The breeds included 10 Domestic Short Hair and one Siamese cat. The median age at surgery was 12.0 years (mean 10.9 years; range, 2–17 years), median bodyweight was 4.6 kg (mean 5.0 kg; range, 4.0–7.0 kg) with a median BCS of 6.0 on the 9‐point scale (mean 5.8; range, 5.0–7.0). Orthopedic examination was performed a median of 5.0 days after the first reported signs of lameness (mean 18.9 days; range, 0–131 days). The hindlimb lameness was classified following the Reinke's time‐dependent classification (Table 2)^18^ as 7.7% acute (*n* = 1), 53.8% subacute (*n* = 7) and 30.7% chronic (*n* = 4). All cats exhibited complete plantigrade stance with associated lameness (Figure 3A), and varying degrees of muscle atrophy of either the right, left or both hindlimbs. Injuries involved the right hindlimb in 53.8% of limbs (*n* = 7) and the left hindlimb in the other 46.2% of limbs (*n* = 6). One cat had simultaneous bilateral lesions, and another one presented with a right AT lesion 3 months after treatment of a left AT lesion with a Novaten implant. Two cats presented with an open skin wound over the injured AT that was treated and fully healed prior to the implant surgery. One cat had the contralateral hindlimb amputated a few years prior to the AT injury. Imaging was performed to confirm the diagnosis and exclude concomitant tarsal injury. All limbs had preoperative radiographs. Three cats had an additional ultrasonography examination and one cat with a bilateral lesion had MRI (Figure 4) and ultrasonography examinations of the AT.

**TABLE 2:** Reinke's time‐dependent classification.^18^

Acute	<2 days between the injury and surgery.
Subacute	2–21 days between injury and surgery.
Chronic	>21 days between injury and surgical treatment.

### 3.2 Surgery and perioperative time

Achilles tendon injuries were classified after opening of the tendon sheath using the classification developed by Meutstege for AT injuries in dogs (Table 3)^19^. A complete (type 1) rupture of the AT was present in 7/13 (53.8%) limbs and the other 6/13 (46.2%) presented with a partial (type 2c) tendon rupture with gastrocnemius tendon (GT) avulsion and preservation of the superficial digital flexor tendon. The lesion was located at the tendino‐osseous junction in all cases. All 13 limbs underwent surgical treatment using the Novaten 2000 implant. No intraoperative complications occurred. Postoperative external coaptation was used in 9/13 (69.2%) limbs. Rigid coaptation using a splint was used in 4/13 (30.8%) limbs, whereas non‐rigid coaptation using a soft‐padded modified Robert‐Jones bandage was used in 5/13 (38.5%) limbs. No external coaptation was used in the remaining 4/13 limbs. Median durations of postoperative external coaptation and activity restriction were 6 weeks (mean 4.1 weeks; range, 0–8 weeks) and 8 weeks (mean 8.2 weeks; range, 4–16 weeks) respectively.

**TABLE 3:** Meutsege's classification for AT injuries in dogs.^19^

Type I	Complete AT rupture
Type II	Lengthened AT‐system with 3 subtypes: Musculo‐tendinous rupture AT rupture with an intact paratenon GT avulsion with an intact superficial digital flexor tendon
Type III	Tendinosis and/or peritendinitis

Abbreviation: AT, Achilles tendon; GT, gastrocnemius tendon.

### 3.3 Complications and outcome

**FIGURE 5:**
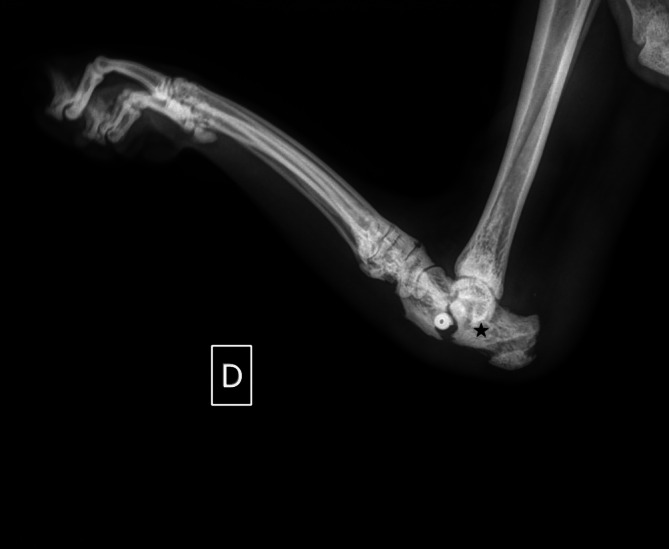
Lateral radiographs at recheck 38 days postoperatively showing a calcaneal fracture at the site of the interference screw. The star points at the ideal location the interference screw, just proximal and caudal to the talocalcaneal central joint.

**FIGURE 6:**
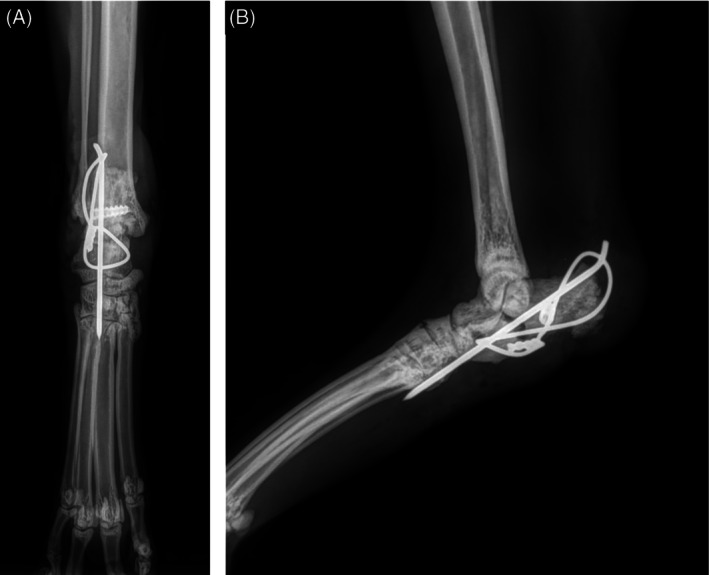
Cranio‐caudal (A) and lateral (B) views of immediate postoperative radiographs following calcaneal fracture repair.

The median duration of the follow‐up period was 15.4 months (mean 19.3 months, 9.5–33 months). Immediate postoperative follow‐up (0–3 months) was available for all 13 limbs with a median follow‐up time of 43 days (mean 45.1 days, 15.0–65.0 days). During this period, 2/13 limbs (15.4%) developed postoperative complications. Lameness scores were grade 0 in 4/13 (30.8%) limbs, 5/13 (38.4%) limbs had grade 1 lameness, 2/13 (15.4%) had a grade 2 lameness and 2/13 (15.4%) had a grade 3 lameness (Figure 3A). One cat presented a grade 3 lameness associated with a return to a plantigrade stance and pain on flexion and extension of the tarsus 38 days after surgery. A calcaneal fracture was present on recheck radiographs at the site of the interference screw (Figure 5). Postoperative radiographic findings were consistent with calcaneal fracture secondary to interference screw malposition, placed more distally than described in the technique which led to an increase in the load on a screw that was inserted in a thinner part of the calcaneum. Osteosynthesis of the calcaneum was performed using a tension‐band wire (Figure 6). The cat showed complete recovery after revision surgery. Another cat presented with a grade 3 lameness which was associated with an open wound and a surgical site infection (SSI). At re‐examination, the limb exhibited a semi‐plantigrade stance with marked tarsal hyperflexion, suggesting partial loss of functional stability of the construct. Recheck radiographs confirmed absence of the interference screw, consistent with implant failure. This complication was therefore classified as a catastrophic complication. Conservative management with systemic antibiotics was elected due to the cat's overall condition and age, but persistent severe lameness was noted at short‐term follow‐up. Short‐term follow‐up (3–6 months) was available for 11 out of the 13 limbs with a median follow‐up time of 116.0 days (mean 125.6 days, 92.0–183.0 days). The orthopedic examination was performed by the referral vet or the orthopedic surgeon. Three cats (4 limbs) presented with a persistent grade 1 lameness including the two cats with bilateral ruptures (one had a bilateral grade 1 lameness and the other a unilateral grade 1 lameness). The other seven cats had lameness score 0 with normal posture (Figure 3B). Long‐term follow‐up at more than 1 year after surgery was available for 12 out of 13 limbs with the completion of a FMPI questionnaire. The median time between surgery and completion of the FMPI questionnaire was 18.5 months (mean 20.4 months, 12.1–33 months). The median age of the cats at this time was 13.0 years (mean 12.4 years, 5–19 years) and the median FMPI score was 1.5 (mean 4.00, 0–27.0) indicating satisfactory long‐term outcome (Figure 3E). One case was diagnosed with a multifocal central nervous system lymphoma 9.5 months after surgery and died shortly afterwards. At the time of neurologic diagnosis, the owner did not report any anomaly related to the AT, but the FMPI could not be performed due to neurologic symptoms (i.e., non‐ambulatory tetraparesia, ataxia). Overall, lameness had significantly improved in all limbs over time compared to preoperative, with two limbs still presenting mild and moderate lameness (Figure 3A). These include one cat that had simultaneous bilateral ruptures who still presented a grade 1 lameness on one side and the cat with previous SSI. The posture for most cats improved from the preoperative plantigrade stance. (Figure 3B). A total of 50% of limbs had an abnormal posture at the first follow‐up control, which resolved over time in all but one limb (Figure 3B). The persistent abnormal posture at long‐term follow‐up occurred in the cat that developed a surgical site infection with subsequent loss of the interference screw. In this case, the limb remained semi‐plantigrade and functionally compromised, consistent with failure of the repair construct. During manipulation of the joint in flexion and extension, few signs of pain were observed in a minority of limbs (Figure 3C,D). Follow‐up evaluations revealed no differences in lameness, pain, or posture between cats with or without rigid coaptation, or according to its duration. Similarly, FMPI scores at the final follow‐up did not differ.

## 4 DISCUSSION

Achilles tendon reconstruction using the Novaten implant is a viable alternative treatment for cats, possibly eliminating the need for external fixation. A total of 11 out of 12 limbs with complete follow‐up achieved successful functional recovery. Achilles tendon rupture can be categorized as acute resulting from trauma, or chronic resulting from degenerative tendinopathy that weakens the tendon.^20^, ^21^ Atraumatic lesions accounted for 11/13 (84.6%) of our sample which is slightly higher than previously reported in other cases series.^2^, ^22^ In a 21‐cat population, Cervi et al. reported type IIc and I lesions with 48% of atraumatic incidences.^2^ In Häußler et al., 66 AT injuries were described including 71.4% of atraumatic injuries classifying as type IIc lesions and only 9.5% were complete ruptures of the AT.^22^ Type I, IIa, IIb and III injuries were not reported in this study. In humans, spontaneous ruptures are attributed to repetitive stress and sedentary lifestyle–related tendon changes.^22^ The etiology of atraumatic AT rupture in veterinary patients remains unclear, though histopathological studies in dogs have demonstrated fibrovascular proliferation near reactive bone, supporting a degenerative mechanism that may explain bilateral involvement.^2^ Two cats in this series presented with bilateral rupture with one having a reported simultaneous rupture on both sides and had injury of one side after the other 3 months apart. In both studies, the low number of limbs does not allow for any strong conclusion.

AT injury is uncommon in cats^2^, ^22^ and tends to affect older individuals, particularly in non‐traumatic lesions.^2^, ^22^ While a female predominance is noted in humans^22^ reports in cats are inconsistent: Cervi et al.^2^ found 86% female cats, Häußler et al.^22^ reported 59.1% females. In the series herein, the sex distribution was more balanced with 54.5% males and 45.5% females. Age appears to influence injury type, with atraumatic ruptures more frequent in older patients.^22^ Similar to humans,^8^ degenerative cellular changes associated with aging may weaken tendon structure. In this study, the overall median age was 12 years, increasing to 13 years in atraumatic lesions, which is consistent with the previous statement. Although body condition score (BCS) was inconsistently reported in prior studies,^2^, ^22^ heavier cats may place greater strain on the AT.^8^ In our series, the mean BCS was 6/9. However, without any strict comparison to the general population and with a limited number of limbs, no conclusion can be drawn regarding overweight as a risk factor for AT rupture in this study.

Previous retrospective studies on surgical management of AT rupture reported 41.3% (26/63)^8^ and 33% (7/21)^2^ short‐term complication rates. In the short‐term period, the overall complication rate in this series was low (15.4%) which likely reflects the absence of external skeletal fixation for joint immobilization. Cervi et al. reported that most of the short‐term complications were due to the immobilization technique with only one complication resulting from the repair itself.^2^ In the study by Häußler et al., the authors reported that at least 75% of all minor complications and at least 40% of all major complications were attributable to the immobilization technique.^8^ Avoidance of an external fixator for postoperative limb immobilization also eliminates the need for a second anesthetic event for fixator removal. Complications associated with non‐rigid external coaptation have also been reported, including edema, pyodermatitis, pressure sores, and decreased range of motion.^23^, ^24^ These complications are of particular concern in cats, which often exhibit poor tolerance to external coaptation.^25^, ^26^ In the study by Cervi et al., none of the cats managed with external coaptation (*n* = 5) developed either minor or major complications.^2^ In contrast, Häußler et al. reported that 10 of 21 cats (47.6%) experiencing minor complications had been treated with external coaptation. Among these cats, five (50%) developed bandage‐related complications during short‐term follow‐up, specifically pressure sores at the calcaneus.^8^ In the present study, the marked variability in duration of postoperative external coaptation likely reflects the novelty of the technique and the absence of standardized postoperative recommendations at the time of case management. As participating surgeons were using the implant for the first time in cats, decisions regarding the use and duration of coaptation were based on individual clinical judgment and prior experience with conventional Achilles tendon repair. This explains the broad range observed and limits firm conclusions regarding the necessity of immobilization. The four limbs managed without external coaptation exhibited recovery and clinical outcomes comparable to those of the nine limbs treated with external coaptation. This may suggest that a benefit of this technique is avoiding any postoperative immobilization, but further prospective, comparative studies are required to investigate this. Long‐term success was observed in 12/13 limbs (92.3%) in this series which is similar to the other reported techniques with a success rate between 84% and 94%.^23^ In dogs, documented postoperative complication rates are between 39.5% and 46.5% which is comparable to those in the studies by Cervi et al. and Häußler et al. However, in dogs, immobilization technique did not seem to significantly affect the complication rate or the final functional outcome.^2^, ^4^, ^8^ Calcaneal fracture was a major complication that could be directly related to the procedure. The absence of a standardized protocol for interference screw placement may predispose to malposition, particularly given the limited dimensions of the feline calcaneus. Based on anatomical considerations, we suggest the optimal screw position be immediately caudal and proximal to the talocalcaneal central joint (Figure 5). Future biomechanical investigations are warranted to objectively determine the ideal screw placement and to characterize the resultant mechanical forces acting upon the construct.

The infection rate was low in this study despite the use of non‐resorbable braided material. However, this finding must be interpreted cautiously given the small sample size, which may underestimate the true incidence of SSI. In the affected case, infection resulted in loss of the interference screw and failure of the anchoring construct, leading to persistent semi‐plantigrade stance and severe lameness with permanent impact on functional outcome. This cat did not have any open wound before surgery, he was 17 years old at the time of surgery and 19 years of age at final follow‐up. Older age could have altered recovery owing to degenerative state of the tendon, weak immune system, and decreased ability to heal properly^27^ which could also explain, in part, his higher FMPI score compared to the other cases (FMPI score of 27). Published data regarding infection rates following Achilles tendon repair in cats remain limited. The two larger feline case series available did not report a high incidence of implant‐related infection, with infections mostly due to the immobilization technique rather than the construct itself.^2^, ^8^ As this represents the first feline case series evaluating this specific implant, larger prospective studies will be necessary to more accurately define the true SSI risk in cats. The avoidance of rigid postoperative immobilization in the present study may have contributed to the low overall complication rate, potentially reflecting the biomechanical properties of the implant that provide load‐sharing support compared to conventional suture‐based repairs.^11^, ^12^, ^13^ Complete absence of coaptation was reported in 4/13 (30.7%) limbs without any complication or loosening of the implant. One case had an amputation on the contralateral hindlimb and did not show any signs of hyperflexion during the removal of the modified Robert Jones at the 2‐week postoperative recheck appointment. The overall repair allows management of challenging cases including in case of complete tendinous rupture direct from to the calcaneus, or avulsion fracture with small or comminuted bone fragments. The technique can also be used where tendon ends have retracted, excessive inadequate scar tissue have formed, and muscles have atrophied or retracted.

Cervi et al. and Häußler et al. reported significantly higher short‐term complication rates, primarily related to the immobilization technique, with no long‐term consequences. The long‐term outcomes in these studies were similar to those of the present study. Long‐term return to function might be at least equivalent with the use of the Novaten implant compared with other techniques. More cases would be needed to strengthen this assessment. The absence of tarsal immobilization may contribute to a better short‐term recovery. Mechanical loading and early return to function has been shown to improve the remodeling of the tendon and its return to a functional state in humans.^21^, ^27^, ^28^ Complete immobilization of tendon repairs can lead to a reduction in repair strength.^20^, ^21^, ^27^, ^28^ On the other hand, cast immobilization has been shown to enhance healing of tendon to bone when compared to complete tendon unloading.^28^

Several limitations should be considered when interpreting the results of this study. The retrospective and multi‐institutional design introduced inherent variability in surgical technique, perioperative management, and postoperative care, potentially influencing the incidence of complications and functional outcomes. The small sample size limited statistical power and prevented robust evaluation of associations between factors such as lesion type, use of coaptation, and clinical recovery. Additionally, the absence of a control group precludes direct comparison between the present technique using the UHMWPE implant with other repair methods or immobilization techniques. Variation in postoperative coaptation protocols and reliance on owner‐reported outcomes via the FMPI introduce potential bias and subjectivity in the long‐term assessment of limb function. Advanced imaging modalities such as ultrasonography or MRI were not routinely employed to objectively evaluate tendon healing or implant integration. Finally, as cases were drawn primarily from referral centers, selection bias may limit generalizability to the wider feline population.

In conclusion, use of the Novaten 2000 implant may represent a promising alternative for AT reconstruction in cats. This study suggests comparable long‐term results, a notably reduced complication rate during the perioperative period, and superior compatibility with the tendon's natural healing process, eliminating tarsal immobilization‐related issues. Biomechanical studies, along with prospective or retrospective studies involving larger sample sizes, would be beneficial for comparing AT reconstruction techniques in greater detail.

## AUTHOR CONTRIBUTIONS

Bohin C, DVM: Contributed to the design of the study, compiled data for four cases, interpreted all data, provided intraoperative photographs, drafted and revised the manuscript. Reinsch G, DVM: Compiled data for three cases, helped in the reference research and revised the manuscript. Vedrine B, DVM, DESV: Was responsible for the surgical management of two cases, compiled data for these cases and revised the manuscript. Ragetly G, DVM, PhD, DACVS (Small Animal), DECVS: Was responsible for the surgical management of one case, compiled data for this case, provided intraoperative photographs and revised the manuscript. Buttin P, DVM, DESV: Was responsible for the surgical management of one case, compiled data for this case and revised the manuscript. Venter FJ, DVM, MSc‐Agric, BVSc, MBA, MMedVet (Small Animal Surgery): Was responsible for the surgical management of one case, compiled data for this case and revised the manuscript. Deacon J, BVM&S, CertSAS: Was responsible for the surgical management of one case, compiled data for this case and revised the manuscript. Bernard F, DMV, IPSAV, DESV, DECVS: Contributed to the design of the study, was responsible for the surgical management of three cases and revised the manuscript. All authors provided a critical review of the manuscript and endorse the final version. All authors are aware of their respective contributions and have confidence in the integrity of all contributions.

## CONFLICT OF INTEREST STATEMENT

The authors declare no conflicts of interest related to this report.
